# Gender, residence, and socioeconomic differences in the relationship of nutrition literacy with vegetable and fruit intake in adults

**DOI:** 10.3389/fnut.2025.1606315

**Published:** 2025-08-06

**Authors:** Xi Tian, Xiaoting Hu, Yuhui Sun, Huaqing Liu

**Affiliations:** School of Public Health, Bengbu Medical University, Bengbu, Anhui, China

**Keywords:** nutrition literacy, vegetable, fruit, adults, differences

## Abstract

**Background:**

Intake of vegetable and fruit is vital for long-term health outcomes. Nutrition literacy (NL) is an important influencing factor of dietary habits. This study aims to explore the relationship between NL and vegetable and fruit intake.

**Methods:**

A cross-sectional survey was conducted in Bengbu, China. Ordinal logistic regression was utilized to explore the correlation between NL and intake of vegetable and fruit, reporting odds ratios (*OR*) and 95% confidence intervals (*CI*).

**Results:**

Approximately 71.0, 40.4, and 40.0% of participants reported consistent consumption of dark-colored vegetables, light-colored vegetables, and fruit, respectively. Individuals with the highest NL were 53% more likely to consume dark-colored vegetables (*OR* = 1.53, 95% *CI*:1.13–2.09), 34% more likely to consume light-colored vegetables (*OR* = 1.34, 95% *CI*: 1.03–1.75), and 200% more likely to consume fruit (*OR* = 3.00, 95% *CI*: 2.29–3.94) than those with the lowest NL. In subgroup analyses, the association of NL with dark-colored vegetables intake was observed among females, urban residents, non-farmers and those with high monthly income. Additionally, the relationship between NL and light-colored vegetables intake was revealed among non-farmers. Conversely, the correlation of NL with fruit intake was exhibited in those had a monthly income < 1,000 RMB and had a monthly income of 1,000–3,000 RMB.

**Conclusion:**

This study found a positive relationship between NL and vegetable and fruit intake. However, this relationship exhibited variations based on gender, residence, and socioeconomic status. Public health practitioners should tailor nutrition intervention programs to improve vegetable and fruit consumption among adults in the studied region, with a particular focus on females, urban residents, and individuals with high monthly incomes.

## 1 Introduction

Vegetable and fruit constitute a vital part of the human diet, offering an array of nutrients and bioactive constituents, such as minerals, vitamins, phytochemicals, carotenoids, and dietary fibers (1), which have multiple benefits in reducing the risk of health-related diseases. Consuming specific types of vegetable and fruit may offer protection against depressive symptoms (2). Particularly, dark-colored plant foods exhibit significant antiaging properties (3). Consuming light-colored vegetables has been correlated with a decreased risk of fall-related fragility fractures (4). Additionally, fruit is recognized for their low-to-moderate energy density, making them advantageous for weight management (5).

Many countries have established dietary recommendations on vegetable and fruit; however, insufficient consumption of vegetable and fruit is still a common problem around the world. For instance, only 12% of European population meets the recommended daily intake of vegetable and fruit (6). Similarly, young adults in Australia failed to meet these consumption guidelines (7). In Nepal, the majority of residents consume fewer than the recommended daily intake of five or more servings of vegetable and fruit (8). Moreover, the consumption of fruit among the Chinese labor force remains below the global average, and the continuing decline in vegetable consumption is a key concern (9, 10). Inadequate consumption of vegetable and fruit intake may be associated with an increased risk of all-cause mortality, cardiovascular disease and cancers (11–13). Studies have indicated that globally, low vegetable consumption accounted for 44.6 million disability-adjusted life years (DALYs), while low fruit consumption resulted in 72.6 million DALYs (14).

The challenge of enhancing vegetable and fruit consumption within the population persists. This multifaceted issue has been examined in recent studies, with determinants including external environmental interventions, socioeconomic and demographic factors, as well as personal dietary behaviors (6, 15, 16). Diet habits, as one of vital factors, is associated with food choices. A study has suggested that students’ unhealthy eating habits predispose them towards selecting a detrimental diet, including fast foods and sugary beverages, while simultaneously showing a deficiency in the consumption of vegetable, fruit, and legume (17). Eating behaviors is influenced by some factors including nutrition knowledge, nutrition education and attitudes. Nutrition education can provide sufficient information on nutrition and healthy diet, which is commonly applied to different people (18, 19). Previous research showed that consumers with a good nutrition education or nutrition knowledge base are more likely to make better dietary behaviors (20). However, inconsistent evidence indicates that individuals with greater nutrition knowledge or awareness do not necessarily exhibit improved eating habits (21). Furthermore, food-related attitudes may affect the selection of food (22). Hence, it is important to explore the key factors influencing the consumption of vegetable and fruit in order to develop targeted interventions.

Nutrition literacy (NL) is the capability to obtain, understand and evaluate nutrition information to choose appropriate foods and make nutritional decisions (23). Recent studies have displayed that NL promotes the selection of high-quality dietary patterns, with individuals demonstrating high levels of NL exhibiting a greater consumption of healthier food options (24, 25). Another study showed that a high of NL is indicative of high consumption of low-fat dairy products, vegetables, nuts and seeds, olive oil, and soya products. There is a positive relationship between individuals with greater NL and increased consumption of vegetable and fruit (26). However, there is a paucity of research investigating the relationship between NL and vegetable and fruit intake in China. This study aims to fill this gap by examining this relationship among Chinese adults.

## 2 Methods

### 2.1 Study design

A cross-sectional study, conducted from May to July 2023 in Bengbu, China, aimed to investigate the association between NL and health among adult individuals. Multi-stage stratified random sampling was employed to select participants. Initially, two urban districts and two rural counties/townships were randomly selected as urban and rural sampling points. Subsequently, two streets and two towns/villages were chosen randomly from each urban districts and rural counties/townships. Finally, 110 households were sampled randomly from each street and town/village. Inclusion criteria including: being aged 18 years or older, possessing clear consciousness, sufficient verbal communication skills, and the ability to complete questionnaires independently or with researcher assistance. Further details have been presented in a previous study (27). A self-designed questionnaire survey was conducted to collect participants’ information on demographic characteristics, socio-economic conditions, lifestyle, diet and health status. Interviews were executed one-to-one and in person by investigators who underwent training in standardized procedures. All participants were advised of the voluntary nature of their participation, and subsequently gave their written informed consent. The study received approval from the Ethical Review Commission of Bengbu Medical University (approval number: 099 from 2021).

Of the original 2,279 participants who completed the interview. 39 (1.7%) were subsequently excluded due to the missing data on nutrition literacy and vegetable and fruit intake. Consequently, 2,240 adult individuals were analyzed in this study.

### 2.2 Nutrition literacy evaluation

The short-form NL self-assessment questionnaire (NL-SF12) was, developed by Liu and colleagues, used to assess participants’ NL (28, 29). The scale comprised 12 items distributed across six dimensions: nutrition knowledge and understanding, obtaining, applying, interactive and critical skills. Each item was scored using a five-point Likert scale, where participants were required to respond to each question. Each statement was evaluated on a five-point Likert scale (Supplementary Table S1), with the categories of strongly disagree, disagree, average, agree, and strongly agree receiving scores of 1, 2, 3, 4, and 5, respectively. The total score for each dimension and the overall NL was calculated as the sum of the individual item scores. A higher score suggests a superior level of NL. For this study, NL was divided into four groups according the quartiles. The NL-SF12 has shown acceptable reliability and validity among the Chinese adults (30, 31). In the present study, the Cronbach’s alpha coefficient of the NL-SF12 was measured at 0.880, exhibiting high internal consistency.

### 2.3 Vegetable and fruit consumption

The Food Frequency Questionnaire (FFQ) was utilized to properly estimate individuals’ dietary consumption, a suitable tool in examining the impact of diet on health and disease (32). The FFQ was constructed based on diet habits and experiences in China. Participants were asked to recall the consumption frequency for each listed food item during the past year. Studies have showed that the FFQ was a reasonably valid instrument for evaluating the dietary intake of foods and nutrients in Chinese adults (33, 34). In this study, the consumption frequency of vegetable and fruit was assessed by three questions (Supplementary Table S2): “How often do you eat dark-colored vegetables?” “How often do you eat light-colored vegetables?” and “How often do you eat fruit?” Responses options ranged from “rarely or never,” “sometimes” and “at least once a month” (occasionally), “at least once a week” (often), and “almost every day” (always).

### 2.4 Covariates

We adjusted for covariates with potential confounding effects: gender (female, male), age (< 60 years, ≥ 60 years), marital status (married, others), place of residence (rural areas, urban areas), educational level (junior high school and below, attained a high school diploma or higher education level), occupation (farmer, non-farmer), monthly income level (< 1,000 RMB, 1000–3,000 RMB, or ≥ 3,000 RMB), smoking history (yes, no) and drinking history (yes, no).

### 2.5 Statistical analysis

Date analyses were performed utilizing SPSS 27.0. Descriptive statistics were used to characterized the distributions of NL and vegetable and fruit intake. Categorical variables were presented as numbers and proportions in this. These variables were compared across different groups using a chi-square test. Additionally, ordinal logistic regression models assessed the association between NL and vegetable and fruit consumption using the lowest level group as a reference. Furthermore, subgroup analyses based on gender and socio-economic status were examined potential disparities in the relationship between NL and vegetable and fruit intake among adults across these characteristics. The results of these models were presented as odds ratio (*OR*) with corresponding 95% confidence intervals (*CI*). A *p-*value less than 0.05, when interpreted two-sided, was deemed to be statistically significant.

## 3 Results

### 3.1 Basic characteristics

Demographic characteristics of the 2,240 participants are presented in Table 1. Of the total sample of 2,240 individuals, 37.5% (*n* = 840) were under the age of 60, 38.4% (*n* = 861) was males, 46.0% (*n* = 1,031) resided in urban areas, 77.8% (*n* = 1743) were married, 30.0% (*n* = 671) had attained a high school diploma or higher education level, 68.9% (*n* = 1,544) were non-farmers, 39.9% (*n* = 894) of the adults reported an income level of less than 1,000 RMB per month, 27.0% (*n* = 605) had a history of smoking, 35.7% (*n* = 799) had a history of alcohol consumption.

**Table 1:** Relationships between demographic characteristics of study subjects and frequency of vegetable and fruit intake.

Variables	*N* (%)	Frequency of dark-colored vegetables intake *n* (%)			*χ^2^*	Frequency of light-colored vegetables intake *n* (%)			*χ^2^*	Frequency of fruit intake *n* (%)			*χ^2^*
		Occasionally	Often	Always		Occasionally	Often	Always		Occasionally	Often	Always	
Total	2,240 (100.0)	75 (3.3)	575 (25.7)	1,590 (71.0)		271 (12.1)	1,063 (47.5)	906 (40.4)		507 (22.6)	837 (37.4)	896 (40.0)	
Age (years)					2.56				0.76				62.75 ***
<60	840 (37.5)	23 (2.7)	227 (27.0)	590 (70.2)		104 (12.4)	406 (48.3)	330 (39.3)		127 (15.1)	300 (35.7)	413 (49.2)	
≥60	1,400 (62.5)	52 (3.7)	348 (24.9)	1,000 (71.4)		167 (11.9)	657 (46.9)	576 (41.1)		380 (27.1)	537 (38.4)	483 (34.5)	
Gender					1.02				2.79				23.55 ***
Male	861 (38.4)	30 (3.5)	211 (24.5)	620 (72.0)		99 (11.5)	395 (45.9)	367 (42.6)		220 (25.6)	351 (40.8)	290 (33.7)	
Female	1,379 (61.6)	45 (3.3)	364 (26.4)	970 (70.3)		172 (12.5)	668 (48.4)	539 (39.1)		287 (20.8)	486 (35.2)	606 (43.9)	
Place of residence					15.20 ***				24.59 ***				202.27 ***
Urban areas	1,031 (46.0)	18 (1.7)	267 (25.9)	746 (72.4)		91 (8.8)	533 (51.7)	407 (39.5)		146 (14.2)	312 (30.3)	573 (55.6)	
Rural areas	1,209 (54.0)	57 (4.7)	308 (25.5)	844 (69.8)		180 (14.9)	530 (43.8)	499 (41.3)		361 (29.9)	525 (43.4)	323 (26.7)	
Marital status					18.27 ***				15.22 ***				0.30
Married	1743 (77.8)	52 (3.0)	416 (23.9)	1,275 (73.1)		199 (11.4)	802 (46.0)	742 (42.6)		390 (22.4)	653 (37.5)	700 (40.2)	
Others	497 (22.2)	23 (4.6)	159 (32.0)	315 (63.4)		72 (14.5)	261 (52.5)	164 (33.0)		117 (23.5)	184 (37.0)	196 (39.4)	
Educational level					11.96 **				4.54				179.91 ***
Junior high school and below	1,569 (70.0)	66 (4.2)	401 (25.6)	1,102 (70.2)		203 (12.9)	727 (46.3)	639 (40.7)		448 (28.6)	624 (39.8)	497 (31.7)	
High school diploma or higher education	671 (30.0)	9 (1.3)	174 (25.9)	488 (72.7)		68 (10.1)	336 (50.1)	267 (39.8)		59 (8.8)	213 (31.7)	399 (59.5)	
Occupation					1.77				6.68 *				150.03 ***
Farmer	696 (31.1)	28 (4.0)	183 (26.3)	485 (69.7)		95 (13.6)	303 (43.5)	298 (42.8)		235 (33.8)	308 (44.3)	153 (22.0)	
Non-farmer	1,544 (68.9)	47 (3.0)	392 (25.4)	1,105 (71.6)		176 (11.4)	760 (49.2)	608 (39.4)		272 (17.6)	529 (34.3)	743 (48.1)	
Monthly income level (RMB)					11.96 *				9.78 *				214.57 ***
<1,000	894 (39.9)	39 (4.4)	242 (27.1)	613 (68.6)		121 (13.5)	399 (44.6)	374 (41.8)		304 (34.0)	378 (42.3)	212 (23.7)	
1,000–3,000	684 (30.5)	25 (3.7)	175 (25.6)	484 (70.8)		87 (12.7)	324 (47.4)	273 (39.9)		125 (18.3)	252 (36.8)	307 (44.9)	
≥3,000	662 (29.6)	11 (1.7)	158 (23.9)	493 (74.5)		63 (9.5)	340 (51.4)	259 (39.1)		78 (11.8)	207 (31.3)	377 (56.9)	
Smoking history					1.41				1.58				23.88 ***
Yes	605 (27.0)	23 (3.8)	146 (24.1)	436 (72.1)		77 (12.7)	274 (45.3)	254 (42.0)		176 (29.1)	226 (37.4)	203 (33.6)	
No	1,635 (73.0)	52 (3.2)	429 (26.2)	1,154 (70.6)		194 (11.9)	789 (48.3)	652 (39.9)		331 (20.2)	611 (37.4)	693 (42.4)	
Drinking history					2.53				4.38				3.80
Yes	799 (35.7)	22 (2.8)	217 (27.2)	560 (70.1)		100 (12.5)	399 (49.9)	300 (37.5)		188 (23.5)	313 (39.2)	298 (37.3)	
No	1,441 (64.3)	53 (3.7)	358 (24.8)	1,030 (71.5)		171 (11.9)	664 (46.1)	606 (42.1)		319 (22.1)	524 (36.4)	598 (41.5)	

* *p* < 0.05, ** *p* < 0.01, *** *p* < 0.001.

As detailed in Table 1, the intake frequency of dark-colored vegetables was 3.3% for those who occasionally consume them, 25.7% for frequent consumers, and 71.0% for those who always consume them. Urban residents, being married, individuals with a high school diploma or higher education level, and those with a monthly income exceeding 3,000 RMB demonstrated significantly higher frequency of dark-colored vegetables consumption compared to their counterparts. The intake frequency of light-colored vegetables was 12.1% for those who occasionally consume them, 47.5% for frequent consumers, and 40.4% for those who always consume them. A rural residency, being married, and being a farmer were associated with more frequent light-colored vegetables consumption. Furthermore, the intake frequency of fruit was 22.6% for those who occasionally consume them, 37.4% for frequent consumers, and 40.0% for those who always consume them. Individuals under the age of 60, females, and urban residents had higher fruit intake frequency. Additionally, individuals with a high school diploma or higher education level, non-farmers, and those with a monthly income exceeding 3,000 RMB were associated with more frequent fruit consumption.

### 3.2 Frequency of consumption of vegetable and fruit according to NL

**Figure 1:**
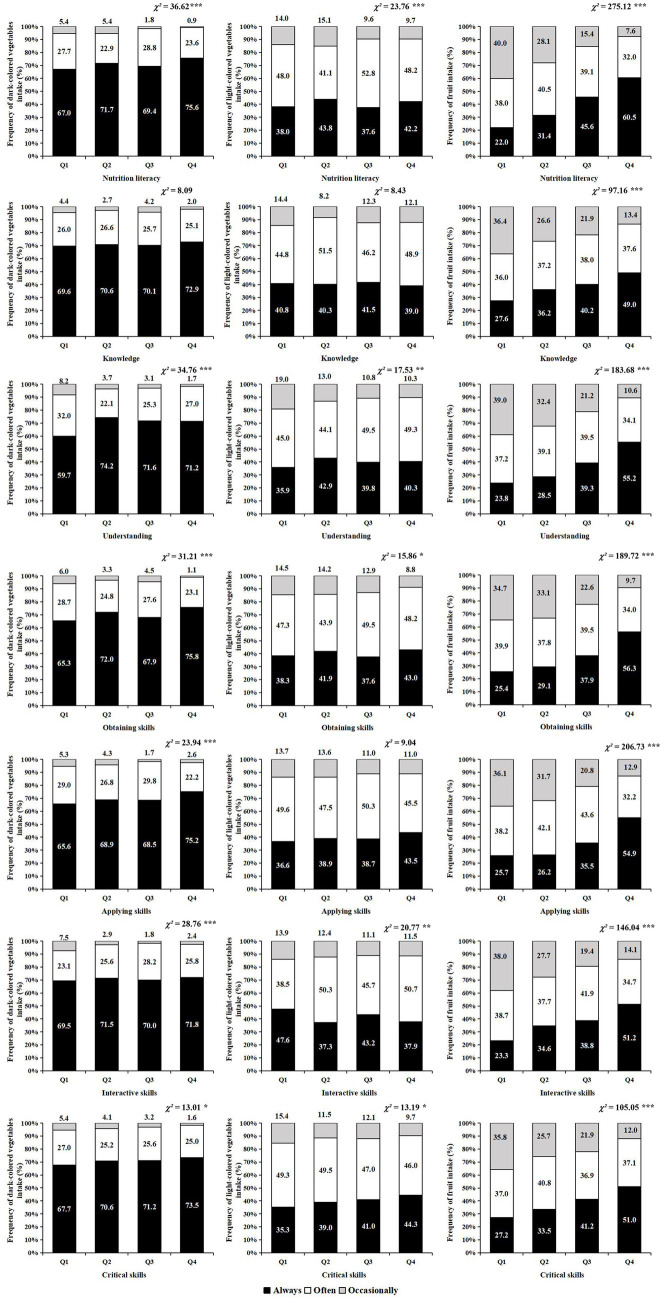
Frequency of dark-colored vegetables, light-colored vegetables and fruit intake according to quartiles of nutrition literacy. * *p* < 0.05, ** *p* < 0.01, *** *p* < 0.001.

Frequencies of dark-colored, light-colored vegetables and fruit intake among adults according to quartiles of NL are presented in Figure 1. A higher level of total NL was significantly related to higher consumption of dark-colored vegetables, light-colored vegetables, and fruit. This association was found in the areas of understanding, obtaining skills, interactive skills and critical skills. However, knowledge of nutrition exhibited a significant correlation solely with the fruit intake, but did not maintain a statistically significant relationship with the intake of dark-colored and light-colored vegetables. Moreover, applying skills showed a significant association with the intake of dark-colored vegetables and fruit, but did not show a significant association with the intake of light-colored vegetables.

### 3.3 Association of NL with the frequency of vegetable and fruit intake

Table 2 shows the *OR* for intake frequency of vegetable and fruit for the NL quartiles. After potential confounders adjustment, individuals with the highest NL were 53% more likely to consume dark-colored vegetables (*OR* = 1.53, 95% *CI*:1.13–2.09), 34% more likely to consume light-colored vegetables (*OR* = 1.34, 95% *CI*: 1.03–1.75), and 200% more likely to consume fruit (*OR* = 3.00, 95% *CI*: 2.29–3.94) than those with the lowest NL. Within the six dimensions examined, this positive relationship was consistently observed across understanding, obtaining skills and applying skills. Interestingly, knowledge was just positively related to intake frequency of fruit (*OR* = 1.82, 95% *CI*:1.43–2.32). Both interactive skills and critical skills were significantly positively associated with the frequency of fruit intake (*OR* = 2.34, 95% *CI*:1.85–2.95 and *OR* = 1.79, 95% *CI*:1.39–2.30, respectively) and light-colored vegetables (*OR* = 0.76, 95% *CI*:0.60–0.96 and *OR* = 1.59, 95% *CI*:1.24–2.05, respectively), but not with the intake frequency of dark-colored vegetables (*OR* = 1.13, 95% *CI*:0.87–1.48 and *OR* = 1.29, 95% *CI*:0.97–1.71, respectively).

**Table 2:** Association of nutritional literacy with the frequency of vegetable and fruit intake.

Variables	Frequency of dark-colored vegetables intake *OR* (95%*CI*)	*P-*value	Frequency of light-colored vegetables intake *OR* (95%*CI*)	*P-*value	Frequency of fruit intake *OR* (95%*CI*)	*P-*value
Nutrition literacy (Ref. =Q1)	–		–		–	
Q2	1.23 (0.95–1.59)	0.114	1.19 (0.94–1.49)	0.147	1.51 (1.21–1.89)	<0.001
Q3	1.13 (0.86–1.50)	0.383	1.13 (0.88–1.45)	0.324	2.04 (1.60–2.62)	<0.001
Q4	1.53 (1.13–2.09)	0.007	1.34 (1.03–1.75)	0.032	3.00 (2.29–3.94)	<0.001
Knowledge (Ref. =Q1)	–		–		–	
Q2	1.04 (0.75–1.43)	0.836	1.12 (0.84–1.49)	0.438	1.46 (1.10–1.94)	0.009
Q3	0.96 (0.74–1.24)	0.748	1.06 (0.84–1.33)	0.636	1.50 (1.20–1.88)	<0.001
Q4	1.13 (0.85–1.49)	0.402	1.00 (0.79–1.28)	0.971	1.82 (1.43–2.32)	<0.001
Understanding (Ref. =Q1)	–		–		–	
Q2	2.00 (1.46–2.76)	<0.001	1.44 (1.07–1.94)	0.016	1.20 (0.90–1.61)	0.209
Q3	1.70 (1.22–2.36)	0.002	1.41 (1.04–1.91)	0.028	1.44 (1.07–1.94)	0.017
Q4	1.60 (1.13–2.26)	0.008	1.45 (1.05–1.99)	0.022	2.02 (1.48–2.76)	<0.001
Obtaining skills (Ref. =Q1)	–		–		–	
Q2	1.45 (1.08–1.94)	0.014	1.14 (0.87–1.48)	0.345	1.15 (0.89–1.49)	0.287
Q3	1.16 (0.87–1.55)	0.302	1.06 (0.82–1.37)	0.673	1.22 (0.95–1.57)	0.120
Q4	1.72 (1.27–2.34)	<0.001	1.40 (1.07–1.83)	0.015	1.98 (1.52–2.59)	<0.001
Applying skills (Ref. =Q1)	–		–		–	
Q2	1.21 (0.91–1.60)	0.185	1.12 (0.87–1.44)	0.374	1.03 (0.81–1.32)	0.800
Q3	1.18 (0.86–1.61)	0.305	1.15 (0.87–1.53)	0.313	1.42 (1.07–1.87)	0.014
Q4	1.59 (1.21–2.08)	<0.001	1.39 (1.10–1.77)	0.007	2.25 (1.77–2.86)	<0.001
Interactive skills (Ref. =Q1)	–		–		–	
Q2	1.17 (0.88–1.55)	0.289	0.74 (0.58–0.95)	0.019	1.45 (1.13–1.85)	0.003
Q3	1.08 (0.79–1.47)	0.625	0.90 (0.68–1.18)	0.445	1.68 (1.28–2.19)	<0.001
Q4	1.13 (0.87–1.48)	0.361	0.76 (0.60–0.96)	0.021	2.34 (1.85–2.95)	<0.001
Critical skills (Ref. =Q1)	–		–		–	
Q2	1.15 (0.81–1.64)	0.440	1.24 (0.91–1.69)	0.172	1.29 (0.95–1.76)	0.105
Q3	1.16 (0.91–1.48)	0.238	1.34 (1.08–1.67)	0.009	1.36 (1.09–1.69)	0.006
Q4	1.29 (0.97–1.71)	0.085	1.59 (1.24–2.05)	<0.001	1.79 (1.39–2.30)	<0.001

The OR (95% CI) was calculated by the ordinary logistic regression adjusting of age, gender, place of residence, marital status, educational level, occupation, monthly income level, smoking history and drinking history. The Q1 group is referred as the reference group.

### 3.4 Subgroup analyses

As detailed in Table 3, a positive relationship was observed between NL and the frequency of dark-colored vegetables only in individuals who were females (*OR* = 1.54, 95% *CI*:1.03–2.31), urban residents (*OR* = 2.01, 95% *CI*:1.24–3.25), non-farmers (*OR* = 1.93, 95% *CI*:1.32–2.82) and those with a monthly income exceeding 3,000 RMB (*OR* = 3.90, 95% *CI*:1.98–7.70). Furthermore, this positive correlation of NL with the frequency of light-colored vegetables was observed only in individuals with non-farmers (*OR* = 1.58, 95% *CI*:1.14–2.20). Notably, the positive relationship between NL and intake frequency of fruit was observed regardless of gender, place of residence, educational level, and occupation. However, this relationship was found only in adults with a monthly income level of less than 1,000 RMB (*OR* = 3.74, 95% *CI*:2.32–6.02) and those earning between 1,000 and 3,000 RMB per month (*OR* = 3.64, 95% *CI*:2.25–5.90), but not in those with a monthly income exceeding 3,000 RMB (*OR* = 1.33, 95% *CI*:0.71–2.48).

**Table 3:** Subgroup analyses of associations between nutritional literacy and frequency of vegetable and fruit intake.

Subgroups	Variables	Frequency of dark-colored vegetables intake *OR* (95%*CI*)	*P-*value	Frequency of light-colored vegetables intake *OR* (95%*CI*)	*P-*value	Frequency of fruit intake *OR* (95%*CI*)	*P-*value
Gender							
Male	Nutrition literacy (Ref. =Q1)	–		–		–	
	Q2	1.52 (1.00–2.31)	0.050	1.03 (0.71–1.50)	0.866	1.50 (1.41–2.15)	0.028
	Q3	1.52 (0.98–2.37)	0.061	1.00 (0.68–1.48)	0.994	1.66 (1.13–2.43)	0.010
	Q4	1.57 (0.96–2.55)	0.070	1.41 (0.92–2.16)	0.114	2.66 (1.74–4.07)	<0.001
Female	Nutrition literacy (Ref. =Q1)	–		–		–	
	Q2	1.08 (0.78–1.50)	0.651	1.29 (0.97–1.73)	0.085	1.50 (1.12–2.00)	0.006
	Q3	0.92 (0.64–1.33)	0.663	1.25 (0.91–1.73)	0.174	2.30 (1.66–3.19)	<0.001
	Q4	1.54 (1.03–2.31)	0.036	1.34 (0.95–1.89)	0.098	3.17 (2.22–4.51)	<0.001
Place of residence							
Urban areas	Nutrition literacy (Ref. =Q1)	–		–		–	
	Q2	1.44 (0.89–2.35)	0.141	1.12 (0.73–1.74)	0.600	1.30 (0.85–1.99)	0.224
	Q3	1.37 (0.86–2.17)	0.187	1.10 (0.71–1.64)	0.711	1.88 (1.25–2.84)	0.003
	Q4	2.01 (1.24–3.25)	0.005	1.35 (0.88–2.06)	0.170	2.61 (1.71–4.00)	<0.001
Rural areas	Nutrition literacy (Ref. =Q1)	–		–		–	
	Q2	1.12 (0.83–1.53)	0.453	1.21 (0.92–1.58)	0.172	1.60 (1.23–2.10)	<0.001
	Q3	1.01 (0.70–1.46)	0.957	1.19 (0.86–1.65)	0.282	2.03 (1.47–2.80)	<0.001
	Q4	1.27 (0.82–1.96)	0.286	1.39 (0.95–2.02)	0.087	3.24 (2.22–4.71)	<0.001
Educational level							
Junior high school and below	Nutrition literacy (Ref. =Q1)	–		–		–	
	Q2	1.18 (0.90–1.54)	0.235	1.17 (0.92–1.48)	0.200	1.50 (1.19–1.89)	<0.001
	Q3	1.15 (0.84–1.57)	0.382	1.20 (0.91–1.57)	0.203	2.01 (1.53–2.64)	<0.001
	Q4	1.42 (0.98–2.06)	0.066	1.23 (0.89–1.69)	0.205	3.13 (2.27–4.31)	<0.001
High school diploma or higher education	Nutrition literacy (Ref. =Q1)	–		–		–	
	Q2	1.48 (0.53–4.19)	0.457	1.28 (0.52–3.16)	0.589	1.44 (0.58–3.55)	0.431
	Q3	1.12 (0.44–2.89)	0.812	1.02 (0.45–2.34)	0.959	1.97 (0.85–4.55)	0.115
	Q4	1.84 (0.71–4.74)	0.209	1.46 (0.64–3.33)	0.371	2.60 (1.13–5.99)	0.025
Occupation							
Farmer	Nutrition literacy (Ref. =Q1)	–		–		–	
	Q2	1.01 (0.70–1.48)	0.941	1.31 (0.94–1.83)	0.108	1.59 (1.15–2.21)	0.006
	Q3	0.90 (0.56–1.47)	0.681	0.98 (0.64–1.50)	0.916	1.46 (0.98–2.27)	0.061
	Q4	1.01 (0.54–1.88)	0.981	0.79 (0.46–1.36)	0.401	3.52 (2.05–6.04)	<0.001
Non-farmer	Nutrition literacy (Ref. =Q1)	–		–		–	
	Q2	1.46 (1.02–2.09)	0.038	1.10 (0.80–1.52)	0.556	1.46 (1.07–1.99)	0.017
	Q3	1.38 (0.97–1.97)	0.075	1.27 (0.92–1.74)	0.146	2.27 (1.65–3.11)	<0.001
	Q4	1.93 (1.32–2.82)	<0.001	1.58 (1.14–2.20)	0.006	2.94 (2.11–4.10)	<0.001
Monthly income level (RMB)							
<1,000	Nutrition literacy (Ref. =Q1)	–		–		–	
	Q2	0.98 (0.71–1.37)	0.925	1.21 (0.91–1.63)	0.197	1.54 (1.15–2.05)	0.003
	Q3	0.85 (0.56–1.29)	0.444	1.10 (0.75–1.60)	0.630	2.16 (1.48–3.16)	<0.001
	Q4	1.36 (0.78–2.38)	0.286	1.50 (0.93–2.42)	0.096	3.74 (2.32–6.02)	<0.001
1,000 ~ 3,000	Nutrition literacy (Ref. =Q1)	–		–		–	
	Q2	1.31 (0.78–2.21)	0.308	0.95 (0.60–1.51)	0.831	1.85 (1.17–2.94)	0.009
	Q3	1.44 (0.86–2.42)	0.165	1.02 (0.65–1.61)	0.927	2.61 (1.65–4.11)	<0.001
	Q4	1.37 (0.80–2.34)	0.253	1.09 (0.68–1.74)	0.735	3.64 (2.25–5.90)	<0.001
≥3,000	Nutrition literacy (Ref. =Q1)	–		–		–	
	Q2	3.20 (1.55–6.64)	0.002	1.61 (0.84–3.05)	0.149	0.73 (0.39–1.40)	0.315
	Q3	2.15 (1.12–4.10)	0.021	1.39 (0.76–2.52)	0.282	0.89 (0.48–1.64)	0.708
	Q4	3.90 (1.98–7.70)	<0.001	1.73 (0.94–3.18)	0.078	1.33 (0.71–2.48)	0.369

The OR (95% CI) was calculated by the ordinary logistic regression adjusting of age, gender, place of residence, marital status, educational level, occupation, monthly income level, smoking history and drinking history. The Q1 group is referred as the reference group.

## 4 Discussion

This study explored the relationship between NL and intake frequency of vegetable and fruit among Chinese community residents. Our findings revealed a positive association between NL and frequencies of vegetable and fruit intake. However, only two in five individuals consistently consumed vegetable and fruit. This was consistent with previous studies that only one in five individuals consumed green leafy vegetables daily, and less than one in five individuals consumed fruit daily (35). Another study showed that mere 12.1% of participants consumed vegetable and fruit at the recommended level (36). Moreover, research reported that Chinese adults aged 50 and above had a higher average consumption of vegetable compare to fruit (37). These results implied that the intake of vegetable and fruit remains relatively low in Chinese community adults. Enhancing NL could potentially encourage adults to adopt healthier dietary behavior and promote their consumption of vegetable and fruit intake.

Our study showed that individuals with high NL are more likely to consistently consume vegetable and fruit. Previous study showed that health literacy, as a predictor of healthy behaviors, was linked to vegetable and fruit consumption (38). Individuals with greater levels of NL were more likely to choose healthier diets (26). Furthermore, people with sufficient NL tended to consume fewer unhealthy foods and preferred nutrient-dense foods (39). Hence, higher NL may improve consumption of vegetable and fruit by promoting healthy dietary habits. This correlation was noted across various dimensions, including understanding, obtaining skills, and applying skills. Notably, the relationship between nutrition-related knowledge and dietary behaviors was intricate, and shaped by the interplay of multiple factors (40). This study revealed a noteworthy phenomenon- a positive correlation between nutritional knowledge and fruit intake, while such an association was not observed with vegetable consumption. This could potentially be attributed to the fact that, although individuals may be aware of the benefits of vegetable consumption or the dietary guidelines set forth by these recommendations, they struggle to effectively translate this knowledge into actionable behaviors (41). Additionally, improving culinary skills, including the ability to read nutritional labels and plan nutritious meals, could potentially affect individuals’ healthy attitudes towards healthy eating. This, in turn, may encourage a greater preference for a variety of vegetable and fruit (42, 43). This process may need the ability of critical and interactive skills. This study showed that critical and interactive skills have a significant impact on frequencies of light-colored vegetables and fruit intake, although they did not exhibit a significant correlation with dark-colored vegetables intake. This could potentially be attributed to the fact that dark-colored vegetables are abundant with polyphenols, often characterized by astringency, bitterness, and pungency (3, 44). Sensory attributed such as bitterness and unattractive color can negatively affect vegetable acceptance (45). Furthermore, individuals’ cognitive development was associated with their perceptions and preferences for vegetable, which were deeply ingrained by their familial environment (46). Future research should explore additional potential factors.

To further investigate the association between NL and vegetable and fruit intake, stratification analyses were conducted according to demographic characteristics. Our results showed a positive correlation between NL and the intake frequency of dark-colored vegetables specifically in females. Previous research indicated that females tend to possess superior knowledge regarding healthy eating habits and nutritional knowledge, and this is likely due to their traditionally dominant role in food purchasing and meal preparation (47). As key players in household food and nutrition dynamics, females can affect their eating behaviors as well as their family’s food consumption and overall health management. Empirical evidence underscored the importance of promoting healthy behavioral practices among females (48, 49). On the other hand, due to gender roles and social responsibilities, males often do not participate as actively in cooking as females. Their dietary choices, influenced by their interest in healthy eating and time constraints, may lead to them to opt for more convenient foods, despite their knowledge of nutrition. Furthermore, factors such as appearance, flavor, and odor can deter males from consuming dark-colored vegetables (22). These findings thus provide a robust foundation for nutrition interventions targeted towards females.

The results also displayed a significant association between NL and dark-colored vegetables intake among urban residents, non-farmers, and those with higher monthly incomes. Dietary habits were shaped by the interplay of various factors, notably socioeconomic status (50). Education, occupation and income served as strong predictors of health behavior and dietary choices (51). Typically, urban residents, who were predominantly non-farmers, had more opportunities to access nutritional education and knowledge (52). Conversely, the high cost of vegetables was a significant barrier to adequate consumption, especially for rural inhabitants (53). Educational level, a pivotal factor correlated with NL, affected food choices by enhancing one’s ability to understand and process nutrition-related information. Individuals with a higher educational levels tended to consume diets recommended by guidelines and exhibit healthier dietary patterns (51, 54). Moreover, education was a reliable predictor of future employment and income. Those with higher educational attainment often possess greater financial resources to invest in their nutritional well-being (55). Occupation and income, moreover, reflected one’s access to food resources. Economic independence from employment may facilitate easier access to nutrition information and the ability to afford health-related products. A higher monthly income was correlated with better food literacy, enabling individuals to afford nutrition courses, receive related education, and choose nutritious foods (54, 56, 57). These were associated with improvement of vegetable. However, our study found no significant differences in educational level across subgroup analyses. We speculated that the ongoing dissemination of nutrition plan and guidance from national dietary guidelines could raise awareness about the benefits of a healthy diet, regardless of educational attainment. Aligning with the above, the association between NL and light-colored vegetables intake was noted among non-farmers. In rural regions, where the preponderance of the labor force was engaged in the agriculture and typically consumes home-grown foods, their primary source of income was singular. Furthermore, due to limitations in purchasing channels, even those aware of the health benefits of vegetable might not consume them regularly (58, 59). Our findings underscored the importance of customized nutritional education and skills training programs designed to enhance vegetable consumption, particularly for non-farmers. We also suggested intervention measures, such as regular dissemination of nutritional knowledge and publication of healthy recipes, to boost intake of dark-colored vegetables among urban residents and those with higher monthly incomes.

Interestingly, our analysis revealed that NL was linked to fruit intake in adults with lower monthly incomes. People with higher income levels often possess greater nutritional knowledge (52). As income levels increase, individuals are more inclined to consciously monitor their diets, prioritize their health status and improve their dietary quality (60). Importantly, these individuals may not be susceptible to external influences and have the financial capability to purchase fruit (61). Hence, nutrition interventions, such as implementing educational activities focused on NL and offering free nutritional seminars, should be tailored to promote fruit intake in individuals with lower monthly income.

Our results emphasized that the need to bolster the multi-dimensional NL to increase the consumption of vegetable and fruit. Nevertheless, the study has certain limitations. Firstly, the cross-sectional design precludes causal inferences. Although relationships are identified, both NL and dietary intake are measured cross-sectionally, suggesting that individuals with healthier diets might perceive themselves as more nutrition-related knowledge, potentially affecting self-reported NL levels. It remains unclear whether higher NL leads to healthier dietary habits or if individuals with better diets seek more nutritional knowledge. Further prospective studies are required to verify their association in the future. Secondly, respondents tend to overestimate their consumption of healthy foods or exaggerate their nutritional knowledge, leading to discrepancies. These discrepancies may be attributed to the study’s reliance on self-reported data rather than observed practices or objective nutritional data. Future research should consider employing more scientific measurements to mitigate such biases. Thirdly, some unmeasured factors, such as cooking skills and food environment, may act as residual confounders, potentially influencing both NL and dietary habits. Future studies should control these variables to help elucidate the complicated relationships. In addition, seasonal variations in vegetable and fruit intake are not taken into account in this study, which may influence the results. Finally, participants are recruited from adults living in one city in Anhui province, and given the disparities in social, cultural, geographic environments, and dietary patterns, it would be inappropriate to generalize the results to other regions. Further research is required to explore the generalizability of these findings across different regions and populations.

## 5 Conclusion

High levels of NL appear to be related to higher intake frequencies of vegetable and fruit, particularly in terms of understanding, acquiring skills, and applying skills. In addition, this relationship varies by gender, place of residence, occupation, and monthly income. Our findings indicate that nutrition education and skill training programs should be launched for non-farmers to promote dark-colored and light-colored vegetable consumption. Moreover, nutrition intervention measures should be applied for females, urban residents, and people with high monthly incomes to encourage dark-colored vegetable consumption in the studied area. The results also highlight that nutrition intervention programs should be considered for low-income people to increase their fruit consumption.

## Data Availability

The raw data supporting the conclusions of this article will be made available by the authors, without undue reservation.
