# Supplementary material for: Gender, residence, and socioeconomic differences in the relationship of nutrition literacy with vegetable and fruit intake in adults
Source: Front Nutr. 2025 Aug 6;12:1606315. doi: 10.3389/fnut.2025.1606315 (PMC12364647; doi:10.3389/fnut.2025.1606315)
Supplement: Supplementary file 1 [file Table_1.docx]

# *Supplementary Material*

# Supplementary Table S1

| **The short-form NL self-assessment questionnaire** | |
| --- | --- |
| Please select the option that best matches your situation. | |
| Appropriate dietary is an important measure to prevent and control chronic diseases such as diabetes and hypertension. | 1. Strongly disagree 2. Disagree 3. Average 4. Agree 5. Strongly agree |
| Compared to frying and grilling, steaming and boiling are healthier ways of cooking. | 1. Strongly disagree 2. Disagree 3. Average 4. Agree 5. Strongly agree |
| I can easily understand the nutritional information delivered by traditional and new media. | 1. Strongly disagree 2. Disagree 3. Average 4. Agree 5. Strongly agree |
| I have a good understanding of expert consensus regarding nutritional information. | 1. Strongly disagree 2. Disagree 3. Average 4. Agree 5. Strongly agree |
| I know where to find healthy diet information. | 1. Strongly disagree 2. Disagree 3. Average 4. Agree 5. Strongly agree |
| I often read nutrition information transmitted through new media (e.g., WeChat and microblogging) or watch nutrition-related program. | 1. Strongly disagree 2. Disagree 3. Average 4. Agree 5. Strongly agree |
| I drink milk or dairy products every day. | 1. Strongly disagree 2. Disagree 3. Average 4. Agree 5. Strongly agree |
| In daily life, I often purchase healthy foods based on nutrition labels. | 1. Strongly disagree 2. Disagree 3. Average 4. Agree 5. Strongly agree |
| I am open to reasonable nutrition and health advice from families or friends. | 1. Strongly disagree 2. Disagree 3. Average 4. Agree 5. Strongly agree |
| If my family members or friends are overweight and enjoy eating high-fat foods, I will encourage them to make dietary changes. | 1. Strongly disagree 2. Disagree 3. Average 4. Agree 5. Strongly agree |
| I can easily tell whether my daily diet is reasonable. | 1. Strongly disagree 2. Disagree 3. Average 4. Agree 5. Strongly agree |
| I can estimate the suitable food intake for maintaining a healthy body weight. | 1. Strongly disagree 2. Disagree 3. Average 4. Agree 5. Strongly agree |

# Supplementary Table S2

| **Food frequency questionnaire** | |
| --- | --- |
| Please recall the frequency of consumption of various foods over the past 12 months. | |
| How often do you eat dark-colored vegetables (e.g., leafy greens, spinach, water spinach, tomatoes, green peppers, carrots, ect.)? | 1. Rarely or never 2. Sometimes 3. At least once a month 4. At least once a week 5. Almost every day |
| How often do you eat light-colored vegetables (e.g., cabbage, radishes, etc.)? | 1. Rarely or never 2. Sometimes 3. At least once a month 4. At least once a week 5. Almost every day |
| How often do you eat fruit (e.g., apples, bananas, grapes, watermelon, etc.)? | 1. Rarely or never 2. Sometimes 3. At least once a month 4. At least once a week 5. Almost every day |
